# The Role of Trace Elements and Minerals in Osteoporosis: A Review of Epidemiological and Laboratory Findings

**DOI:** 10.3390/biom13061006

**Published:** 2023-06-17

**Authors:** Anatoly V. Skalny, Michael Aschner, Ekaterina V. Silina, Victor A. Stupin, Oleg N. Zaitsev, Tatiana I. Sotnikova, Serafima Ia. Tazina, Feng Zhang, Xiong Guo, Alexey A. Tinkov

**Affiliations:** 1 Laboratory of Ecobiomonitoring and Quality Control, Yaroslavl State University, 150003 Yaroslavl, Russia; skalny3@gmail.com; 2 Center of Bioelementology and Human Ecology, Institute of Biodesign and Modeling of Complex Systems, Department of Therapy of the Institute of Postgraduate Education, IM Sechenov First Moscow State Medical University (Sechenov University), 119435 Moscow, Russia; silinaekaterina@mail.ru (E.V.S.); t-sotnikova@yandex.ru (T.I.S.); tazin.re@yandex.ru (S.I.T.); 3 Department of Molecular Pharmacology, Albert Einstein College of Medicine, Bronx, NY 10461, USA; michael.aschner@einsteinmed.edu; 4 Department of Hospital Surgery No. 1, Pirogov Russian National Research Medical University, 117997 Moscow, Russia; stvictor@bk.ru; 5 Department of Physical Education, Yaroslavl State Technical University, 150023 Yaroslavl, Russia; irisha-zip@yandex.ru; 6 City Clinical Hospital n. a. S.P. Botkin of the Moscow City Health Department, 125284 Moscow, Russia; 7 Key Laboratory of Trace Elements and Endemic Diseases, National Health and Family Planning Commission, Health Science Center, School of Public Health, Xi’an Jiaotong University, Xi’an 710061, China; fzhxjtufeng@163.com (F.Z.); guox@mail.xjtu.edu.cn (X.G.)

**Keywords:** osteoporosis, trace elements, bone mineral density, minerals, bone resorption, selenium, zinc, fluoride, strontium

## Abstract

The objective of the present study was to review recent epidemiological and clinical data on the association between selected minerals and trace elements and osteoporosis, as well as to discuss the molecular mechanisms underlying these associations. We have performed a search in the PubMed-Medline and Google Scholar databases using the MeSH terms “osteoporosis”, “osteogenesis”, “osteoblast”, “osteoclast”, and “osteocyte” in association with the names of particular trace elements and minerals through 21 March 2023. The data demonstrate that physiological and nutritional levels of trace elements and minerals promote osteogenic differentiation through the up-regulation of BMP-2 and Wnt/β-catenin signaling, as well as other pathways. miRNA and epigenetic effects were also involved in the regulation of the osteogenic effects of trace minerals. The antiresorptive effect of trace elements and minerals was associated with the inhibition of osteoclastogenesis. At the same time, the effect of trace elements and minerals on bone health appeared to be dose-dependent with low doses promoting an osteogenic effect, whereas high doses exerted opposite effects which promoted bone resorption and impaired bone formation. Concomitant with the results of the laboratory studies, several clinical trials and epidemiological studies demonstrated that supplementation with Zn, Mg, F, and Sr may improve bone quality, thus inducing antiosteoporotic effects.

## 1. Introduction

Osteoporosis is considered as a skeletal disorder characterized by reduced bone strength leading to increased fracture risk [1]. According to the World Health Organization (WHO) diagnostic criteria, osteoporosis is characterized by a 2.5 or more standard deviations of lower bone mineral density than the mean peak bone mineral density (BMD) for healthy adults [2]. Primary osteoporosis is induced by estrogen deficiency in postmenopausal women (Type I) as well as in both ageing men and women (Type II, senile osteoporosis) [3]. Secondary osteoporosis is formed due to a particular pathology or treatment with pharmacological agents [4], similar to that observed in glucocorticoid-induced osteoporosis, type 2 diabetes mellitus, obesity, and systemic inflammatory disease, to name a few [5]. At the same time, rare causes of both primary and secondary osteoporosis have also been identified [6].

The results of a recent meta-analysis demonstrated that the overall prevalence of osteoporosis worldwide is 18.3%, although it is characterized by a high geographic variability, with the highest prevalence in Africa reaching 39.5% of the adult population [7]. In the EU, osteoporotic fractures are considered as the fourth most significant pathology contributing to total disability-adjusted life years after ischemic heart disease, dementia, and lung cancer [8]. The annual economic burden of osteoporosis-related fractures accounts for more than USD 17 billion in the US [9]. It is expected that the number of fractures and osteoporosis-related costs will further increase in the future decades [10].

The pathogenesis of osteoporosis involves the alteration of mechanisms of regulation of bone remodeling. The latter results from increased osteoclast activity leading to bone resorption and impaired osteoblast activity with decreased bone formation [11]. The molecular mechanisms underlying these alterations were shown to involve increased RANKL production and down-regulated OPG secretion with a subsequent decrease in the OPG/RANKL ratio, contributing to the stimulation of osteoclast activity. This mechanism is considered a target for a variety of stimuli, including proinflammatory cytokines, growth factors, and hormones, affecting osteoclast activity. In turn, altered Wnt signaling and the subsequent down-regulation of LRP5 signaling is associated with the inhibition of osteoclastogenesis. In addition, the alteration of this pathway may also contribute to reduced peak bone mass [12]. Given a complex pathogenesis of osteoporosis, various endogenous and exogenous factors modify the risk of osteoporosis [13]. Osteoporosis is considered an age-related disease due to its higher prevalence in advanced-age subjects. Pathogenetic mechanisms involved in senile osteoporosis include ageing-induced alterations in autophagy, iron overload, disturbances in gut microbiota, as well as ageing-associated metabolism dysregulation, altogether resulting in bone marrow mesenchymal stromal cells (BMMSCs) senescence with the subsequent inhibition of osteoporosis and the promotion of adipogenesis [14]. Due to the role of sex steroids in bone physiology, an age-related decline in endocrine function also contributes to osteoporosis, especially in women, resulting in postmenopausal osteoporosis [15]. One of the leading mechanisms of postmenopausal osteoporosis involves estrogen deficiency, which contributes to increased RANKL production and suppression of OPG secretion, altogether resulting in osteoclast activation. In addition, a lack of stimulatory effect of estrogen on growth factor production and a subsequent osteoblast differentiation also contribute to reduced bone formation [16].

Dietary factors including Ca and vitamin D intake as the key regulators of bone health have a significant impact on osteoporosis risk [17]. Calcium (Ca) and phosphorus (P) are the key minerals composing inorganic bone matrix as calcium hydroxyapatite [Ca_10_(PO_4_)_6_(OH)_2_], and the less abundant octacalcium phosphate [Ca_8_H_2_(PO_4_)_6_·5H_2_O] [18]. Therefore, the homeostasis of these minerals is essential for bone formation and functioning. Ca and P metabolism is strictly regulated by the parathyroid hormone (PTH) and 1,25-dihydroxyvitamin D (1,25(OH)_2_D) [19]. The role of Ca and vitamin D as key nutrients for bone health have been widely discussed in the context of osteoporosis in a number of excellent reviews [20,21]. Although Ca is essential for bone health, a systematic analysis of the available data demonstrates that populations from developing countries with lower Ca intake are characterized by a lower risk of osteoporotic bone fractures as compared to developed countries [22]. Correspondingly, it has been demonstrated that the association between increased Ca intake and bone mineral density is clinically irrelevant [23]. These observations demonstrate that micronutrients other than Ca and vitamin D play an important role in osteoporosis [24].

Patients with osteoporosis are characterized by a higher incidence of micronutrient deficiency [25], whereas an improvement in micronutrient intake may result in bone-protective effects against osteoporosis. A recent meta-analysis by Feng et al. (2021) demonstrated that dietary patterns including micronutrient intake are associated with the incidence of osteoporosis [26]. The role of micronutrient deficiency in osteoporosis is also confirmed by observations on postoperative osteoporosis in subjects undergoing bariatric surgery with sleeve gastrectomy and Roux-en-Y-gastric bypass, characterized by impaired micronutrient intake [27]. Previous studies demonstrate that higher serum essential trace element levels (Zn, Cu, Fe) are associated with a lower risk of osteoporosis [28]. An inverse association between trace element and mineral intake and osteoporosis is mediated by the role of those in the modulation of bone physiology targeting the main cell types, osteoblasts, osteocytes, and osteoclasts [29,30].

At the same time, the distinct mechanisms which govern the effects of trace elements and minerals on bone physiology and osteoporosis pathogenesis have yet to be elucidated, and epidemiological findings have been contradictory. Therefore, the objective of the present study was to review recent epidemiological and clinical data on the association between selected minerals and trace element effects with osteoporosis as well as to discuss the molecular mechanisms underlying these associations.

## 2. Magnesium (Mg)

Mg is an essential mineral that shares certain similar chemical properties with Ca, being considered as an essential factor of bone health [31], while adequate Mg intake and homeostasis was shown to be protective against osteoporosis [32]. The results of the meta-analysis demonstrated significantly reduced serum Mg levels in osteoporotic postmenopausal women [33], although this association was country-specific, being significant in European but not Asian populations [34]. In addition, a recent systematic review and meta-analysis showed a significant positive relationship between dietary Mg intake and hip BMD values in older adults [35], corroborating the observed negative association between Mg intake and osteoporosis [36]. The results from a large prospective study demonstrated that higher dietary Mg intake is associated with a reduced risk of future osteoporotic fractures in American adults [37]. Mg supplementation was also shown to reduce bone turnover in Turkish osteoporotic postmenopausal women [38]. In agreement with data on dietary intake, low serum Mg concentration is associated with an increased risk of fractures in middle-aged men from the Kuopio Ischemic Heart Disease cohort [39].

Mg was shown to promote osteogenic differentiation of mesenchymal stem cells through a variety of mechanisms [40], including the up-regulation of the Wnt/β-catenin pathway [41], as well as of BMP-2 [42] and BMP-6 expression [43]. Mg-promoted osteoblast proliferation and differentiation was also associated with increased extracellular signal-regulated kinase (ERK) and glycogen synthase kinase-3 beta (GSK3β) phosphorylation [44]. The activation of the phosphatidylinositol-3 kinase (PI3K)/Akt serine/threonine kinase (Akt) pathway may also underlie the stimulatory effect of Mg on osteoblast differentiation and adhesion [45]. Notch1 signaling may be involved in the Mg-induced osteogenic differentiation of mesenchymal stem cells [46]. It is also notable that Mg deficiency-associated inhibition of osteoblast differentiation was associated with increased inducible nitric oxide (NO) synthase (iNOS) up-regulation with subsequent NO overproduction [47].

Mg was also shown to promote osteoblast motility, resulting in the increased infiltration of osteoblasts in Mg-containing scaffolds [48]. The increased mobility of osteoblasts upon Mg exposure was also associated with the relocalization of zona-occludens 1 tight junction protein into cytoplasm [49]. Mg also promoted intercellular gap junction communication [50].

Due to the beneficial effects of Mg on osteogenesis, Mg-containing biomaterials were considered as potential agents for bone regeneration [51].

At the same time, high Mg levels can inhibit osteoblast differentiation at least partially due to the alteration of intracellular Ca^2+^ levels [52]. Correspondingly, 0.5–2.0 mM Mg increased extracellular matrix mineralization, whereas higher doses were found to be inhibitory [53].

In a coculture of osteoblasts and osteoclasts, Mg significantly reduced osteoclast differentiation in parallel with osteoblastogenesis stimulation [54], indicative of the inhibitory effect of Mg on bone resorption, especially in proinflammatory conditions. Specifically, Mg lithospermate B ameliorated LPS-induced bone resorption through the inhibition of RANKL/RANK-dependent osteoclastogenesis [55]. Correspondingly, the impact of Mg on inflammation-induced bone resorption was shown to be mediated by the Mg-induced reduction in IκB degradation and the subsequent down-regulation of NF-κB signaling in parallel with the inhibition of NFATc1 mRNA and protein expression [56].

The antiresorptive effect of Mg was demonstrated in Mg-deficient conditions. Specifically, Mg deficiency was associated with reduced bone mineral density due to inflammation-induced increase in osteoclast activity along with reduced osteoblast differentiation [57], being in agreement with earlier findings by Rude et al. (2003) [58]. Reduction in dietary Mg by 25% in rats resulted in lower trabecular thickness and reduced bone volume, which may be associated with inflammation-associated activation of osteoclastic bone resorption [59]. The up-regulation of RANKL expression along with the down-regulation of OPG expression was considered a key mechanism linking bone resorption and Mg deficiency [60]. Yet, despite a significant increase in osteoclastogenesis upon Mg deficiency, the resorptive activity of these cells was reduced [61]. Ultrastructural analysis demonstrated that Mg-deficient osteoclasts upon OPG stimulation had decreased contact with the endosteal bone surface and the absence of a ruffled border [62]. In turn, Mg overload resulted in increased osteoclast differentiation by vitamin D3, thus reprograming the effect of vitamin D3 on bone remodeling [63], and thus corresponding to an earlier demonstrated biphasic effect of Mg on osteoclast differentiation and activity [64].

Taken together, the existing data demonstrate that osteoporosis is associated with Mg deficiency, whereas Mg intake or systemic levels directly correlate with BMD, being inversely related to osteoporotic fracture risk. Correspondingly, laboratory in vivo and in vitro findings demonstrated that antiosteoporotic effect of Mg is mediated by the up-regulation of BMP-2/6 and Wnt/β-catenin signaling, as well as the activation of PI3K/Akt and ERK and the promotion of GSK3β phosphorylation. RANKL-dependent osteoclastogenesis was also inhibited by Mg, which is tightly associated with OPG production. However, even for Mg that is characterized by a rather wide therapeutic window, high doses were shown to inhibit osteoblastogenesis and increase osteoclast differentiation.

## 3. Selenium (Se)

Selenium (Se) is also an essential factor of bone development and regulation of bone turnover through multiple mechanisms involving selenoproteins [65].

The existing epidemiological data demonstrate that osteoporosis is associated with low Se levels in hair of both Korean men and women with mean ages of 51–54 years old [66]. Lower dietary Se intake was associated with reduced bone mineral density in Brazilian women [67] and a history of bone fractures in postmenopausal women from the National Health and Nutrition Examination Survey (NHANES) (2013–2014) [68], as well as a higher prevalence of osteoporosis in the general middle-aged and older subjects from China [69]. Correspondingly, Se deficiency was shown to be associated with bone mass loss and trabecular separation due to increased bone resorption [70]. Therefore, Se deficiency is considered a risk factor for osteoporosis [71]. Selenoprotein P is considered an essential transporter of Se to bones [72], whereas selenoprotein W was shown to be involved in the regulation of osteoclast differentiation [73].

The association between Se status and bone quality is mediated by the regulatory effect of Se and selenoproteins on bone formation and bone resorption processes. A number of studies demonstrated that the osteogenic effect of Se was associated with the modulation of redox homeostasis due to its antioxidant activity [74,75,76]. Specifically, Se up-regulates the expression of two antioxidant selenoproteins, glutathione peroxidase (GPX) and thioredoxin reductase (TXNRD), resulting in the activation of osteoblastogenesis and the inhibition of osteoclastogenesis [77]. Concomitantly, sodium selenite increased the mRNA expression of osteogenic transcription factors as well as prevented the H_2_O_2_-induced inhibition of osteoblast differentiation through its antioxidant activity and the activation of the Wnt/β-catenin signaling pathway [78], as well as through the inhibition of ERK activation [79]. Another study demonstrated that Se-induced osteogenesis may be mediated by the modulation of redox homeostasis and the activation of the c-Jun N-terminal kinase (JNK)/Forkhead box O3 (FOXO3) pathway rather than alterations in ERK or p38 mitogen-activated protein kinase (MAPK) signaling [80]. Se nanoparticles promoted osteogenic differentiation of mesenchymal stem cells instead of adipogenic lineage through the activation of Smad-dependent BMP signaling [81].

A number of studies demonstrated the protective effects of Se in models of bone damage. Specifically, the activation of the BMP-2/MAPKs/β-catenin pathway was responsible for preventing diabetic osteoporosis by Se [82]. Se-containing nanoparticles were also shown to alleviate dexamethasone-induced osteoporosis [83]. Se treatment prevented ROS overproduction, dysregulation of Ca signaling, and mitochondrial apoptosis in osteoblast cell line exposed to zoledronic acid, bevacizumab, and dexamethasone [84]. When locally introduced with bone cement, Se promoted bone defect repair through the up-regulation of GPX1 expression [85] and the modulation of OPG/RANKL signaling [86]. The lipopolysaccharide (LPS)-induced apoptosis in MC3T3-E1 osteoblast cells was prevented by Se through the up-regulation of PI3K/Akt signaling [87].

Se was shown to inhibit RANKL-induced osteoclasts differentiation from bone marrow-derived monocytes [88]. Selenite was also shown to induce osteoclast apoptosis through the mitochondrial pathway including mitochondrial dysfunction, cytochrome c leakage, and caspase 3 activation [89]. Se nanoparticles also suppressed osteoclastogenesis through the inhibition of interleukin (IL) 6 signaling [90]. Correspondingly, the Se-mediated prevention of osteoblast dysfunction was shown to invoke the inhibition of NF-κB activation with the subsequent down-regulation of IL-6, monocyte chemoattractant protein-1 (MCP-1), cyclooxygenase (COX) 2, and iNOS production [91].

**Figure 1:**
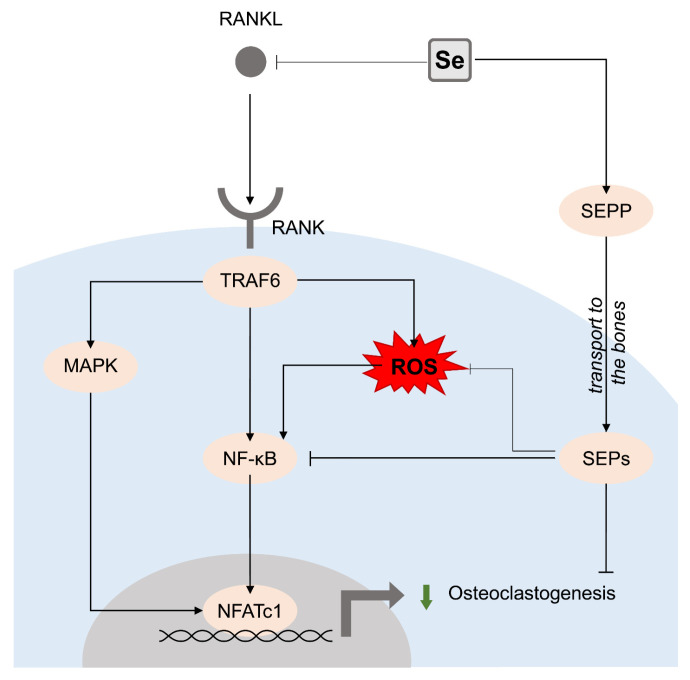
The proposed mechanism of antiresorptive effects of Se. Se is transported to the bone by selenoprotein P (SELENOP), where it is used for synthesis of antioxidant selenoproteins (SEPs) including GPX and TXNRD. Antioxidant activity of selenoproteins inhibits RANKL/RANK/tumor necrosis factor (TNF) receptor-associated factor 6 (TRAF6) signaling-associated ROS overproduction, thus inhibiting activation of redox sensitive NF-κB, resulting in down-regulation of nuclear factor of activated T-cells (NFATc1)-dependent osteoclastogenesis.

Epidemiological data demonstrate that both Se intake and Se status were associated with BMD, reduced bone loss, and lower osteoporosis risk. Being in agreement with the role of Se as an antioxidant or a precursor of antioxidant selenoproteins, its osteoprotective effect of Se against reactive oxygen species (ROS)-induced damage was demonstrated. Beneficial effects of Se may be at least partially mediated by the regulation of the expression of selenoproteins GPX, TXNRD, selenoprotein P (SELENOP), and W (SELENOW). Osteogenic effects of Se were mediated by the stimulation of Wnt/β-catenin and BMP2 signaling associated with ERK, p38 MAPK, PI3K/Akt, and Smad pathways. In turn, Se may reduce bone resorption by inhibiting RANKL-induced osteoclastogenesis and subsequent nuclear factor κB (NF-κB) activation (Figure 1).

## 4. Zinc (Zn)

Zn is an essential factor of bone health that may counteract the development of osteoporosis under different pathological conditions [92]. Zn transport is also critical for physiological bone formation and metabolism [93]. A meta-analysis demonstrated that serum Zn is inversely associated with osteoporosis in both patients with osteoporosis and postmenopausal women, whereas Zn supplementation significantly increases bone mineral density [94]. Plasma Zn levels were shown to be positively associated with vertebra bone mineral density in Turkish postmenopausal women [95]. It is also notable that osteoporosis is associated with higher Zn excretion, which may contribute to Zn deficiency [96].

Zn plays a significant regulatory role in bone mesenchymal stem cell differentiation [97]. Being in agreement with its effect on osteoblast proliferation and differentiation, Zn promoted collagen synthesis [98] and calcium deposition [99]. Correspondingly, Zn deficiency was associated with reduced collagen synthesis and extracellular matrix calcification [100]. At the same time, despite the significant stimulation of osteoblast differentiation at low Zn levels, its overexposure was shown to inhibit bone mineralization [101]. The biphasic effects of Zn may be mediated by Zfp521 signaling [102]. The promotion of osteogenesis by Zn may involve the activation of osteoprotegerin expression due to the up-regulation of phosphoenolpyruvate carboxykinase (PCK) and MAPK/ERK pathways [103]. Another mechanism of Zn-induced osteogenic differentiation may involve the activation of cyclic adenosine monophosphate (cAMP)-protein kinase A (PKA)-cAMP response element-binding protein (CREB) signaling [104].

The role of Zn in bone physiology was also confirmed by the observed adverse effects of Zn deficiency on osteoblasts. Specifically, Zn deficiency is associated with increased mitochondria-mediated apoptosis in osteoblasts [105]. Zn deficiency may be associated with reduced bone mineral density through increased parathyroid hormone production [106,107].

At the same time, Zn deficiency may be associated with the inhibition of both osteoblastogenesis and osteoclastogenesis through the inhibition of Wnt/β-catenin-induced Runt-related transcription factor 2 (Runx2) expression [108] and microphthalmia-associated transcription factor (MITF)-mediated RANK expression, respectively [109].

Laboratory studies demonstrated that, in parallel with the promotion of osteogenic differentiation, Zn treatment is capable of inhibiting osteoclastic and adipogenic differentiation [110] due to the inhibition of NF-κB signaling [111]. Correspondingly, Zn significantly reduced osteoclast-mediated bone resorption [112] via the inhibition of the RANKL/OPG pathway [113,114]. The inhibition of Ca^2+^-Calcineurin-NFATc1 signaling may also be considered as the potential mechanism of Zn-induced suppression of osteoclastogenesis [115].

Given the modulatory role of Zn in the regulation of bone formation and bone resorption, studies have addressed the protective effects of Zn supplementation in animal models of osteoporosis. Specifically, Zn prevented osteoporosis in type 1 diabetic ovariectomized rats through the down-regulation of RANKL signaling [116]. It is also notable that Zn potentiated the antiosteoporotic effect of Ca and vitamin D3 treatment through the down-regulation of the macrophage-colony stimulating factor receptor (M-CSFR) and RANKL signaling [117]. In turn, the promotion of osteogenesis upon Zn supplementation to STZ-diabetic rats was associated with the activation of insulin-like growth factor 1 (IGF-1)/IGF-1 receptor (IGF-1R)/Akt/GSK3β/β-catenin [118].

The osteogenic effect of Zn was used for the construction of biomaterials used as implants [119,120,121] and agents for bone regeneration [122,123,124].

Taken together, the existing epidemiological findings demonstrate that Zn intake and status are positively associated with BMD, being inversely related to osteoporosis risk. Such an association is mediated by the stimulatory effect of Zn on osteoblast proliferation and differentiation through its relationship to Wnt/β-catenin cascade activity as well as the up-regulation of other pathways including MAPK/ERK, cAMP-PKA-CREB, and IGF-1/IGF-1R/Akt/GSK3β/β-catenin signaling. The inhibition of bone resorption by Zn is mediated by the inhibition of RANKL signaling and the promotion of OPG expression, ultimately resulting in the down-regulation of osteoclast differentiation and activity. Given the significant osteoprotective effect of Zn, it was successfully used as a component of biomaterials for implants and bone regeneration.

## 5. Iron (Fe)

Fe plays an essential role in the regulation of bone formation and metabolism [125], whereas the dysregulation of Fe metabolism is associated with osteoporosis [126].

Fe overload severity is associated with osteoporosis in patients with hereditary hemochromatosis [127] and thalassemia major [128]. Age-associated bone Fe accumulation is also associated with reduced bone mass [129]. Therefore, Fe chelators are considered as potential therapeutic agents in osteoporosis [130]. In addition, osteoporosis is associated with reduced serum Fe levels [28], whereas prior Fe-deficiency anemia may be considered as a risk factor for osteoporosis [131], as clearly demonstrated in a national-wide study in Taiwan [132]. These observations demonstrate that both Fe deficiency and overload may be associated with osteoporosis.

In agreement with the observed differential relationship between Fe overload and deficiency with osteoporosis, low (physiological) and high (toxic) doses of Fe exert distinct effects on bone formation and bone resorption. Specifically, a U-shaped relationship was observed between Fe levels and osteoblast activity wherein moderately low Fe doses promoted osteoblast activity and both critically low and high Fe doses inhibited osteoblast functioning due to an increase in ROS production [133]. Both Fe deficiency anemia [134] and Fe overload [135] were shown to affect BMP-2-induced osteoblastogenesis. Finally, it has been demonstrated that physiological Fe levels were essential for osteogenic differentiation, which was significantly impaired by Fe chelation [136]. Fe deficiency was associated with reduced bone mineral density and osteocalcin levels due to the inhibition of renal 1α-hydroxylase activity and a subsequent decrease in 1,25-dihydroxyvitamin D_3_ levels [137].

In turn, high doses of Fe, corresponding to conditions of Fe overload, exert significant toxicity in osteoblasts, affecting bone formation. In particular, Fe overload was shown to reduce alkaline phosphatase activity, type I collagen mRNA and protein expression, as well as deposition of calcium by osteoblasts [138]. Fe overload induced osteoblast apoptosis due to mitochondrial dysfunction and endoplasmic reticulum stress via the phosphorylated eukaryotic initiation factor-2α (eIF2α)/activating transcription factor 4 (ATF4)/CCAAT/enhancer-binding protein (C/EBP) homologous protein (CHOP) pathway [139]. The inhibition of PI3K/AKT/FOXO3a/dual specificity phosphatase 14 (DUSP14) signaling was also shown to be involved in this effect [140]. In addition to apoptosis, Fe overload induced necroptosis in osteoblast cells [141]. One of the recently posited mechanisms for the Fe overload-induced inhibition is ferroptosis [142] associated with iron-responsive element (IRE)/iron regulatory protein 1 (IRP1)-mediated NADPH-activation [143]. The inhibition of Wnt/β-catenin signaling may also be responsible for Fe overload-induced osteoporosis [144], whereas the activation of Wnt signaling may ameliorate the ferroptosis-mediated disruption of osteoblast differentiation [145] with Wnt5a playing a key role [146]. In turn, Fe chelation with deferoxamine (DFO) promoted Wnt5a-dependent osteogenic differentiation through the up-regulation of PI3K/Akt and NFATc1 signaling [147].

ROS generation in osteoblasts exposed to Fe was also promoted by the inhibition of autophagy [116], which may be associated with the mammalian target of rapamycin (mTOR) activation [148].

In addition, serum hepcidin, a negative Fe regulator, was shown to be inversely associated with osteoporosis risk [149] through reduction in Fe levels and ROS generation [150]. Concomitantly, hepcidin was shown to reverse the Fe overload-induced inhibition of osteogenesis [151]. These findings demonstrate that targeting hepcidin should be considered as a potential therapeutic strategy in osteoporosis management [152].

In addition to the adverse effects on osteoblastogenesis and bone formation, Fe overload was associated with aberrant osteoclast activity and subsequent bone resorption. Specifically, Fe overload was shown to promote osteoclastogenesis through the induction of ROS generation [153,154] with RANKL signaling [155] and the subsequent activation of NF-κB signaling [156].

Fe availability and the activation of Fe-uptake proteins with the inhibition of Fe efflux are essential for osteoclast differentiation [157] with Tfr1 playing a key role [158]. At the same time, the impact of hepcidin on osteoclast differentiation remains controversial [159,160].

**Figure 2:**
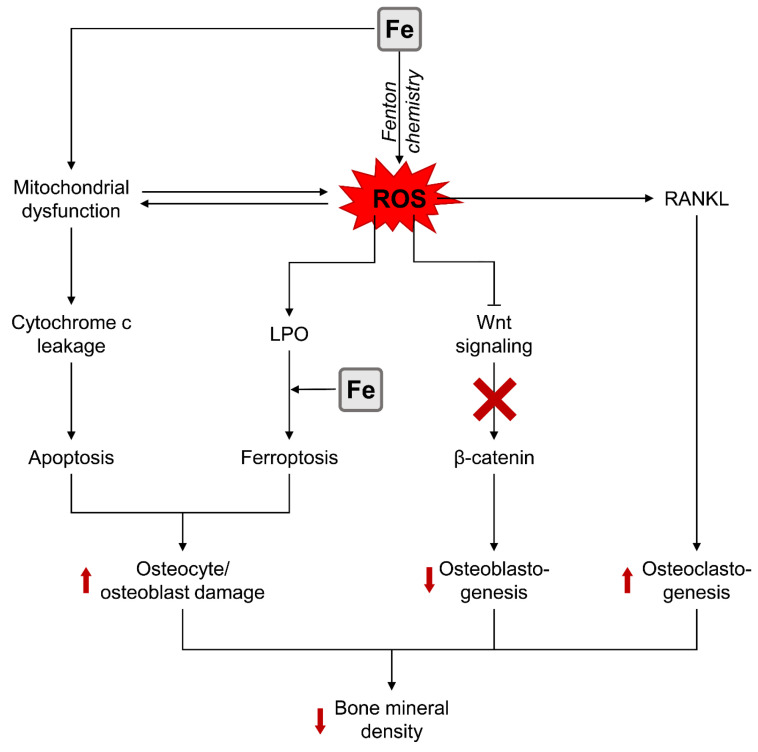
The proposed mechanisms involved in adverse effects of Fe overload in the bone. Fe overaccumulation is associated with increased ROS production via Fenton chemistry as well as Fe-induced mitochondrial dysfunction, which also contributes to ROS generation. In the presence of elevated intracellular Fe levels, ROS induce lipid peroxidation (LPO), in turn triggering ferroptosis. In addition to ferroptosis, Fe-induced mitochondrial dysfunction results in increased cytochrome c leakage and apoptosis, altogether resulting in osteocyte and osteoblast damage. Excessive ROS production due to Fe overload also interferes with canonical Wnt signaling, leading to reduced β-catenin levels and inhibiting osteoblastogenesis. In turn, ROS was also shown to induce excessive RANKL secretion, which promotes osteoclastogenesis. Taken together, Fe overload promotes osteocyte/osteoblast damage, reduced osteoblast differentiation, as well as excessive osteoclastogenesis with induction of bone resorption through ROS-dependent mechanisms.

Recent findings demonstrate that both Fe deficiency anemia and Fe overload in hemochromatosis and thalassemia major may exert adverse effect on bone quality, being in agreement with the observed U-shaped association between Fe exposure and osteoblast activity. Fe deficiency was associated with impaired osteogenic BMP-2 signaling, whereas high-dose Fe exposure exerted a prooxidant effect and induced adverse effects on osteoblast activity through a variety of ROS-dependent mechanisms including mitochondrial dysfunction, endoplasmic reticulum stress, ferroptosis, apoptosis, and necroptosis, associated with the inhibition of BMP-2 and Wnt/β-catenin pathway signaling. Fe-induced ROS overproduction was also related to RANKL-dependent osteoclastogenesis and subsequent bone resorption (Figure 2). Hepcidin, being a negative regulator of Fe metabolism, reversed the inhibitory effect of Fe on osteogenesis.

## 6. Copper (Cu)

Dietary Cu intake was shown to be positively associated with bone mineral density, being inversely related to osteoporosis risk in American adults [161], while osteoporosis patients were characterized as having significantly reduced serum Cu levels [28]. At the same time, the association between Cu status and osteoporosis risk was shown to be non-linear. The analysis of NHANES 2011–2014 data demonstrates that subjects with the lowest serum Cu concentration are characterized by lower BMD values, whereas those at the highest quartile of Cu levels have a higher fracture rate, especially in adult men [162], being indicative of the adverse effect of Cu overexposure on bone health. Correspondingly, the results of the meta-analysis demonstrate that Wilson’s disease, characterized by systemic Cu overload, is directly associated with osteopenia, osteoporosis, and fracture risk in children and middle-aged adults [163]. Therefore, both suboptimal and excessive Cu levels in the organism may increase the risk of osteoporosis. Laboratory studies also demonstrate a U-shaped relationship between Cu exposure and osteogenesis, when low doses of Cu (0.1–1 µM) promote osteogenesis with the increase in bone nodule formation, whereas high doses of Cu (50–100 µM) induce a cytotoxic effect [164].

Cu (50 μM) was shown to promote osteogenic differentiation of mesenchymal stem cells (MSCs) [165] with increased calcium deposition as well as angiogenesis [166]. A number of studies demonstrated the osteogenic effect of Cu addition to different biomaterials. Specifically, the stimulation of osteogenesis by Cu-containing 316L stainless steel was shown to be mediated by Akt activation and Runx2 up-regulation [167]. The doping of porous TiO_2_ coatings with Cu nanoparticles also increased osteoblast proliferation and adhesion along with extracellular matrix mineralization, which may be associated with the stimulation of vascular endothelial growth factor (VEGF) and NO production [168,169]. Cu ion-substituted hydroxyapatite-based titanium dioxide nanotubes were shown to promote osteogenesis through the stimulation of osteoblast adhesion, proliferation, and differentiation [170]. Hypothetically, the beneficial effect of biomaterial-bound Cu as compared to Cu^2+^ ions is likely associated with its lower catalytic activity and reduced ROS generation.

Furthermore, 100–150 μM Cu induced osteoblast damage and dysfunction through the inhibition of the transforming growth factor beta (TGF-β1)/Smad3 pathway [171]. Another study also demonstrated that Cu is capable of inhibiting osteogenic differentiation of bone marrow mesenchymal stem cells with the inhibition of collagen formation [172].

In addition to the modulation of osteoblast differentiation and activity, Cu was shown to reduce osteoclastic bone resorption [173,174]. Correspondingly, Cu supplementation was shown to counteract ovariectomy-induced reduction in bone mineral density [175].

Cu-modified cobalt–chromium particles significantly increased the production of anti-inflammatory cytokines, whereas the expression of proinflammatory cytokines was reduced due to the inhibition of NF-κB, which is also responsible for the down-regulation of osteoclastogenesis as compared to Cu-free particles [176]. Cu-doped titanium alloys inhibited RANKL-induced osteoclastic proliferation with the subsequent inhibition of osteoclast-specific enzymes [177]. At the same time, the impact of Cu^2+^ on osteoclast tartrate-resistant acid phosphatase (TRAP) activity and bone resorption may be different in differentiating and mature osteoclasts [178].

Therefore, despite the adverse effects of Cu overload in Wilson’s disease on bone health, nutritional Cu intake was associated with improved BMD, lower risk of osteoporosis, and fracture rate. The differential effect of Cu supply on osteoporosis risk is mediated by a U-shaped influence of Cu on osteogenesis. While high doses of Cu inhibited osteoblast differentiation through the inhibition of the TGF-β1/Smad3 pathway, low doses of Cu applied as a component of biomaterials promoted the osteogenic differentiation of mesenchymal stem cells via the activation of PI3K/Akt signaling and the stimulation of VEGF and NO production. In addition to the dose, it has been proposed that the beneficial effect of biomaterial-bound Cu may be mediated by its lower catalytic activity and prooxidant effect. Cu-induced prevention of bone resorption is mediated by the inhibition of RANKL-induced osteoclastic proliferation. Therefore, Cu may be considered as a beneficial component of bone biomaterials, although the prooxidant activity of the Cu^2+^ cation may mediate the association between Cu overload and osteoporosis.

## 7. Cobalt (Co)

Co is an essential metal that possesses hypoxia-mimicking activity through the up-regulation of hypoxia-inducible factor 1-alpha (HIF-1α) signaling [179]. Co-based alloys are widely used for bone and joint implants [180], and therefore their impact on bone health is of particular interest. In turn, epidemiological studies on the association between dietary Co deficiency and bone quality are lacking.

A number of studies demonstrated that the incorporation of Co^2+^ into biomaterials significantly improved their osteogenic properties. Specifically, Co-enriched hydroxyapatite significantly improved osteoporotic bone regeneration [181]. Micromolar Co^2+^ embedded into calcium phosphate layers was shown to increase osteoclast differentiation and osteoclastic mineral resorption [182]. At the same time, the effects of Co on osteogenesis were shown to be dose-dependent with low doses (1 ppm) exerting osteogenic, angiogenic, and anti-inflammatory activity, whereas higher doses promoted osteoclastogenesis (5 ppm) and cytotoxicity (>5 ppm) [183]. Low doses of Co (50–100 μmol/L) also significantly increased osteogenesis with the up-regulation of HIF-1α, BMP-2, Runx2 expression and subsequent collagen type 1 production, whereas higher doses of Co suppressed cell proliferation [184]. The doping of tricalcium phosphate scaffolds with Co induced angiogenesis by increasing VEGF expression and human umbilical vein endothelial cells (HUVECs) growth and migration, as well as promoted osteogenesis, whereas excessive Co doping significantly inhibited osteogenesis [185]. These findings corroborate earlier findings by Kim et al. (2002) who demonstrated the HIF-1α-dependent increase in VEGF expression in osteoblast cells [186]. Correspondingly, Co-containing hydroxyapatite at a dose of 1.5% significantly increased osteoblast activity and reduced apoptotic cell death, whereas higher Co content resulted in cytotoxic effects [187].

The effect of Co on osteogenesis was shown to be mediated by its role as HIF-1α inducer. Specifically, the induction of HIF-1α signaling by CoCl_2_ treatment was shown to promote Wnt/β-catenin-mediated osteogenesis [188]. At the same time, Co significantly inhibited osteogenesis in a HIF-1α-dependent manner and increased cell stemness [189,190].

Adverse effects of Co on bone formation were shown to involve a plethora of mechanisms including the inhibition of TGF-β expression [191] and the induction of oxidative stress in osteoblasts [192], as well as inducing necrosis in osteocytes [193]. Co was also shown to inhibit osteoblast migration in addition to the reduction in collagen production [194]. It has been also demonstrated that Co affects collagen matrix formation through the interaction with the hydroxyl group of the carboxylic terminal of the collagen molecule, preventing its stabilization and collagen formation [195].

Co^2+^-induced interference with inflammatory pathways was shown to modulate its effect on osteogenesis. Specifically, the osteogenic effect of Co incorporated with β-tricalcium phosphate was ameliorated in presence of macrophages, which responded to Co-containing tricalcium phosphate with M1 polarization and promoted inflammation [196]. Moreover, it has been demonstrated that, in addition to the inhibition of osteoblast functioning, Co up-regulated the gene and protein expression of IL-8 and MCP-1 [197] and IL-6 production [198], thus promoting an inflammatory response. These Co-induced effects in mature osteoblasts were also accompanied by increased RANKL protein and Toll-like receptor 4 (TLR4) mRNA expression [199], as well as a reduction in the OPG/RANKL ratio, being indicative of a shift to osteoclastogenesis [200].

Concomitantly, existing data demonstrate that the impact of Co on osteoclastic bone resorption is also dose-dependent. Andrews et al. (2011) characterized the effects of Co^2+^ based on its physiological (blood serum) concentrations. It has been demonstrated that, at serum levels, Co treatment led to a mild stimulatory effect on osteoclast formation, whereas higher exposure levels reduced cell number and osteoclast activity [201]. The effect of Co on osteoclast activity was also species-specific. While both CoCl_2_ and Co nanoparticles significantly inhibited osteoclast proliferation and differentiation, low doses of CoNPs increased carbonic anhydrase II (CA II) and cathepsin K mRNA expression [202]. Correspondingly, Co^2+^ incorporated into Ca phosphate bone cement promoted bone resorption by increasing cathepsin K, CA II, and TRAP activity [174]. In turn, Co protoporphyrin, a potent inducer of heme oxygenase 1 (HO-1), was shown to inhibit RANKL-dependent osteoclastogenesis through the modulation of inhibitor of nuclear factor kappa B (IκB), Akt, ERK, JNK, and p38 MAPKs signaling [203].

Taken together, the existing data demonstrate that low-dose Co^2+^ can improve bone health that and its incorporation into biomaterials may potentiate osteogenic effects of the latter, whereas high-dose Co inhibits osteogenesis by promoting cell death, oxidative stress, and inflammation. Given the strong dose-dependence of the effects of Co on osteogenesis and osteoclastogenesis, its introduction into biomaterials needs to be thoroughly regulated, and its release from Co-containing metal implants should be monitored.

## 8. Fluoride (F)

Fluoride is considered as an effective treatment for osteoporosis [204]. The results of the meta-analysis demonstrated that fluoride treatment significantly increases vertebral spine and hip BMD in postmenopausal women, older adults, and patients with various diseases, whereas a reduction in fracture risk was observed only at low daily fluoride intake [205]. Another meta-analysis demonstrated that fluoride was the most effective in increasing BMD in postmenopausal osteoporosis among all agents including bisphosphonate (BP) and vitamin D3 [206]. Concomitantly, elevated serum F levels were not associated with BMD or osteoporotic fractures in American women [207].

Excess fluoride can induce systemic toxicity adverse effects on bone health [208]. However, recent findings demonstrate that community water fluoridation in Korea is not associated with any adverse effects on bone health [209], whereas fluoride exposure from drinking water is not associated with hip fracture risk in a previous meta-analysis [210]. Fluoride exposure at US-specific levels did not have any effect on bone health in adolescents [211]. In Swedish postmenopausal women, higher F intake was associated with increased BMD and hip fracture risk [212], being contradictory to earlier observations in Sweden [213].

Increased F accumulation was shown to result in decreased bone density, bone cortex thinning, reduced bone mineralization [214], as well as altered mechanical properties of the bone with an increase in indentation distances and lower elastic modulus [215].

Fluoride is capable of affecting a plethora of signaling pathways [216] that may underlie its complex effect on bone homeostasis through modulation of proliferation, differentiation, and functioning of osteoblasts and osteoclasts.

Low-dose fluoride treatment was shown to exert a beneficial effect on bone formation through the stimulation of osteoblastogenesis. Specifically, fluoride-induced osteoblast proliferation is dependent on Wnt/β-catenin pathway activation due to down-regulated GSK3β expression [217] or its increased phosphorylation [218]. In addition to GSK3β, the phosphorylation of Akt at Ser473 may also contribute to the activation of Wnt/β-catenin signaling in osteoblasts [219].

The osteogenic effect of fluoride may be associated with the up-regulation of insulin receptor mRNA expression [220]. Correspondingly, the up-regulation of TGFβ1 by fluoride exposure was inhibited in streptozotocin diabetic rats, being indicative of the role of insulin signaling in the osteogenic and osteoclastogenic effects of fluoride [221]. F-induced TGF-β1 expression was shown to mediate the impact of fluoride on autophagy [222]. Low-dose F treatment increased mRNA and protein expressions of connexin 43 and connexin 45 in osteoblasts, whereas high-dose exposure induced an inhibitory effect [223].

Given the role of physiological doses of fluoride in bone functioning, F-releasing chitosan hydrogels [224], strontium-substituted porous apatite microspheres [225], and fluoride-containing bioactive glasses [226] were used for treatment of osteoporosis and bone regeneration. In addition to the osteogenic effect, fluoride-containing bioglasses also possessed bactericidal activity [227].

Fluoride exposure was shown to induce endoplasmic reticulum stress in osteoblasts, which may be involved in the biphasic regulation of osteogenesis. Specifically, F-induced increased protein kinase RNA-like endoplasmic reticulum kinase (PERK) expression [228] due to endoplasmic reticulum stress [229] and unfolded protein response [230] was shown to be associated with osteogenic effect. In contrast, the induction of endoplasmic reticulum stress upon F exposure along with mitochondrial dysfunction triggers apoptosis and autophagy in osteoblast cells [231,232] and osteocytes [233].

High-dose fluoride exposure impaired osteogenesis by inducing osteoblast dysfunction. Fluoride exposure significantly reduced BMP-2 expression [234], which may underlie the inhibitory effect of F on osteoblast differentiation. Fluorosis-induced cell-cycle arrest and apoptosis in osteoblast cells was shown to be counteracted by up-regulated SIRT1 signaling [235] through the induction of autophagy via the sirtuin 1 (SIRT1)-FoxO1-Ras-related protein 7 (Rab7) axis and a SIRT1-FoxO3-Bcl-2 interacting protein 3 (Bnip3) signaling [236]. High-dose fluoride-induced inhibition of osteoblast viability may also be associated with MAPK-mediated Yes-associated protein (YAP) activation [237]. Fluoride also inhibited osteocyte response to mechanical loading due to cytoskeletal alterations [238].

The fluoride-induced effects on bone cells were also tightly associated with the modulation of Ca homeostasis. Specifically, low-dose fluoride significantly reduced PTH-related peptide (PTHrP) expression and increased i[Ca^2+^] in osteoblast cells, whereas high-dose exposure induced inverse changes in parallel with increasing calcium-sensing receptor (CaSR) mRNA and protein levels [239]. An increase in intracellular Ca^2+^ levels and increased osteogenesis upon low-dose fluoride treatment was associated with increased mRNA and protein expression of Cav1.2, the main subunit of L-type voltage-dependent calcium channels [240].

The impact of fluoride on osteogenesis may also involve epigenetic mechanisms. Specifically, fluoride exposure was shown to induce p16 gene hypermethylation [241] and deacetylation [242], resulting in its reduced expression and increased osteoblast proliferation. It has also been demonstrated that high F intake with drinking water is associated with RUNX2 promoter methylation contributing to reduced BMD in women [243]. CALCA (calcitonin-related polypeptide alpha) gene methylation is associated with higher susceptibility to fluoride-induced decrease in BMD in women [244]. Low-dose NaF treatment significantly increased methylguanine methyltransferase (MGMT) and MutL protein homolog 1 (MLH1) gene methylation, resulting in osteoblast proliferation and activation [245]. Fluoride induced DNA hypomethylation of BMP-2 and BMP-7 promoter regions associated with increased protein expression during the development of dental fluorosis [246]. DNA hypermethylation of BMP1, methionyl aminopeptidase 2 (METAP2), matrix metalloproteinase (MMP) 11, and BTB domain and CNC homolog 1 (BACH1) gene promoter was also observed in fluoride-exposed human osteosarcoma cells [247].

**Figure 3:**
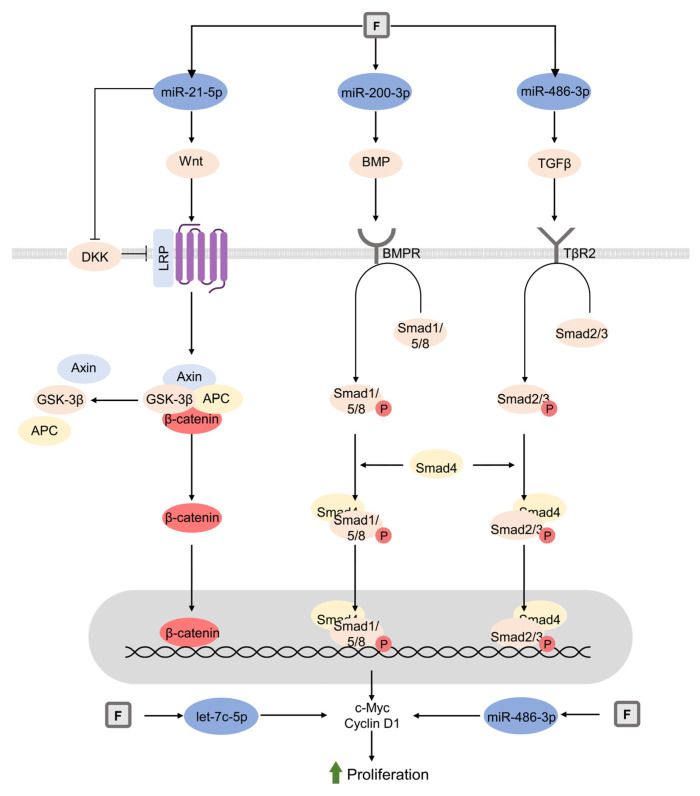
The role of miRNA in mediation of the effects of fluoride in the bone. F-induced modulation of miR-486-3p expression up-regulates cyclin D1 through TGF-β1/Smad2/3, resulting in increased osteoblast proliferation. Let-7c-5p also modulates cyclin D1 upon fluoride exposure. miR-200c-3p was shown to mediate proliferative effects of fluoride via up-regulation of BMP4/Smad pathway. Finally, F-induced increase in miR-21-5p expression promotes Wnt signaling through LRP5/6 and subsequent dissociation of a destruction complex consisting of Axin, GSK3β, and adenomatous polyposis coli (APC), leading to β-catenin accumulation. The impact of miR-21-5p on canonic Wnt signaling may also be mediated by its inhibitory effect on PTEN and DKK. Upward arrow is indicative of stimulation.

The impact of fluoride exposure on osteoblast may be significantly mediated by the modulation of microRNAs (miRNAs) expression [248]. Specifically, miR-486-3p was shown to mediate the up-regulation of cyclin D1 through the TGF-β1/Smad2/3 pathway [249], being in agreement with the role of TGF-β1 in fluoride-induced effects in osteoblasts [250] as evidenced by the F-induced increase in TGF-β receptor 2 (TβR2), smad3, and MAPK expression [251]. MicroRNA (miRNA) let-7c-5p was also shown to be involved in the modulation of cyclin D1 expression by fluoride [252]. Increased miR-21-5p expression upon fluoride treatment was shown to induce canonical Wnt signaling pathway activation with the down-regulation of phosphatase and tensin homolog (PTEN) and Dickkopf WNT Signaling Pathway Inhibitor 2 (DKK2) [253]. miR-200c-3p promoted proliferative effects of fluoride in the SaoS2 cell line via the up-regulation of the BMP4/Smad pathway [254] (Figure 3).

Vitamin D deficiency [255] or Ca excess [256] were shown to aggravate F-induced alterations in bone metabolism. Correspondingly, vitamin D treatment significantly reduced F-induced osteoblast apoptosis [257].

Fluoride was also shown to exert antiresorptive effects due to its impact on osteoclast functioning [258]. However, an inverted U-shaped association between fluoride exposure and osteoclastogenesis was observed with the highest number of osteoclasts upon exposure to medium-dose (50 mg/L F) and a lower number at higher doses, especially in F-free medium [259]. A biphasic effect of F on osteoclast differentiation and activity was shown to be mediated by F-induced TGFβ/TβR1/Smad3 activation [260].

Micromolar fluoride suppressed ageing-induced bone resorption through the inhibition of RANKL signaling, NFATc1, cathepsin K, and MMP-9 expression [261,262,263]. At the same time, the activation of the same RANK-JNK-NFATc1 signaling pathway was shown to underlie the stimulatory effect of high-dose F exposure on osteoclastogenesis [264]. In addition, the up-regulation of interferon gamma (IFNγ) production may also be considered as the potential mechanism of F-induced bone loss in postmenopausal women characterized by a reduction in estrogen, which inhibits IFNγ secretion [265].

**Figure 4:**
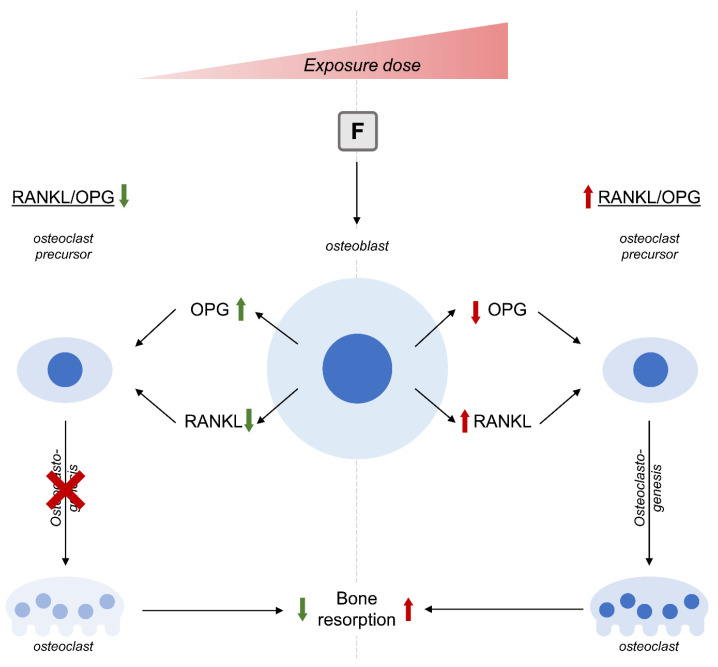
Biphasic effect of fluoride on osteoclastogenesis and bone resorption. Micromolar fluoride concentrations were shown to reduce RANKL production, resulting in decreased RANKL/OPG ratio and down-regulation of osteoclastogenesis, as well as inhibition of bone resorption. In contrast, excessive doses of fluoride up-regulate RANKL production with an increase in RANKL/OPG production, which promotes osteoclast formation and bone resorption. Upward and downward arrows are indicative of stimulation and inhibition, respectively. Green color of the arrows is indicative of positive effect on bone health, whereas red arrows demonstrate effect leading to bone resorption.

Generally, fluoride is considered as an effective agent for the treatment of osteoporosis associated with improvement of BMD. Nonetheless, excessive fluoride intake can exert systemic toxic effects, including adverse effects on bone health, although the latter were not observed at dietary intake. Osteogenic effects of fluoride may be mediated by its stimulatory influence on the Wnt/β-catenin pathway associated with Akt activity, negative regulation of GSK3β activity, up-regulation of TGFβ1 signaling, activation of insulin receptor, as well as endoplasmic reticulum stress modulation. In turn, the negative effect of high doses of fluoride is associated with the inhibition of BMP-2 signaling. The effect of fluoride on osteoclastogenesis was also shown to be biphasic, characterized by the stimulation of RANKL-induced osteoclastogenesis by low-dose F treatment and its inhibition in response to higher doses (Figure 4). Epigenetic mechanisms were also involved in the osteogenic response to fluoride treatment by the modulation of methylation of genes involved in osteoblast differentiation and functioning (RUNX2, CALCA, MGMT, MLH1, BMP2, BMP-7). In addition, F-induced changes in microRNA (miR-486-3p, miRNA let-7c-5p, miR-21-5p, miR-200c-3p) expression may also mediate its effects on osteoblast differentiation and functioning. These findings demonstrate that fluoride is an effective modulator of bone physiology, although its overload may exert adverse effects on bone formation and resorption.

## 9. Strontium (Sr)

Sr is involved in the regulation of bone functioning through a variety of mechanisms [266]. The results of the meta-analysis demonstrated that the administration of Sr ranelate (SrRa) is associated with a 31% and 40% decrease in osteoporotic fractures and vertebral fractures in postmenopausal osteoporosis cases, respectively [267]. Sr ranelate was also shown to promote bone fracture healing [268]. However, SrRa did not improve wrist fracture healing in advanced-age Italians suffering from wrist fracture, while being administered during the acute phase [269].

Long-term SrRa treatment was shown to result in improved BMD over 10 years in women with postmenopausal osteoporosis from the SOTI/TROPOS cohort (Belgium) [270] and patients with thalassemia major-related osteoporosis from Italy [271]. Despite the positive influence on bone health, long-term SrRa administration may significantly increase cardiovascular disease (CVD) risk [272].

In addition, the results of a systematic review demonstrated that Sr supplementation may be considered as an effective agent for the stimulation of implant osteointegration with osteoporotic bones [273]. Correspondingly, a meta-analysis of laboratory studies demonstrated that Sr increases the osteointegration of titanium implant surfaces [274].

Laboratory studies demonstrated that Sr promotes osteogenic differentiation both in mesenchymal and ectomesenchymal bone marrow stromal cells [275], as well as adipose tissue-derived mesenchymal stem cells [276]. The stimulation of osteogenic differentiation by Sr was associated with an increased number of cells in the S and G2/M phases, while maintaining stem cell population at the same size [277]. Moreover, SrRa was shown to inhibit adipocytic but stimulate osteogenic differentiation of bone marrow mesenchymal stem cells, as evidenced by the up-regulation of Runx2 and other genes [278]. In agreement with the observed Sr-induced increase in osteogenic differentiation, Sr increased osteoblast activity, as evidenced by increased type I collagen expression and nodule formation [279].

As for the particular mechanisms, Sr-induced osteoblast proliferation and differentiation associated with ERK phosphorylation and the up-regulation of BMP-2 may be at least partially mediated by CaR activation [280] and the subsequent JAK2/Signal transducer and activator of transcription 3 (STAT3) signaling [281]. The latter was shown to be associated with Akt activation, ultimately resulting in canonical Wnt/β-catenin signaling [282]. In addition, Sr promoted bone regeneration and osteogenesis through the activation of TGF-β/Smad and β-catenin signaling [283], as well as the up-regulation of BMP-2 expression [284].

Sr-induced osteogenesis was also associated with AMP-activated protein kinase (AMPK)-activated autophagy via the phosphorylation of AMPK and a subsequent decrease in mTOR phosphorylation [285]. Similar mechanisms were involved in Sr-induced osteoporotic bone regeneration [286]. It has been also demonstrated that Sr may partially compensate for the lack of calcium for osteogenesis [287].

The epigenetic effects of Sr may be also involved in its osteogenic activity. Specifically, a histone methylase, Setd2 up-regulation was shown to be associated with Sr-induced osteoblast differentiation [288].

Sr was shown to improve bone formation in glucocorticoid-induced osteoporosis [289] through the stimulatory effect of Sr on ERK signaling [290]. Correspondingly, Sr also activated rat sarcoma viral oncogene homolog (RAS) along with increased ERK1/2 and p38 MAPK phosphorylation, ultimately contributing to the osteogenic differentiation of bone marrow mesenchymal stem cells [291].

Analogous to fluoride, Sr-releasing biomaterials including nanoscale cement [292], porous hydroxyapatite bioceramics [293], and bioactive glasses [294] were shown to promote osteogenesis.

Several studies demonstrate that excessive Sr exposure may induce adverse effects on bone formation. Specifically, low doses (0.5–1 μg/mL Sr) reduce bone nodule formation without any impact on bone mineralization, whereas high doses of Sr (20–100 μg/mL) inhibit the mineralization process and hydroxyapatite formation without any alteration of nodule formation [295]. Despite the observed stimulation of osteogenic differentiation in adipose-derived stem cells at lower levels, high-dose Sr exposure induces apoptosis associated with ERK1/2 signaling [296].

In addition to bone formation promotion through the stimulation of osteoblast differentiation and activity [297], Sr was also shown to reduce osteoclast differentiation [298], resulting in decreased bone resorption. Specifically, the Sr-induced up-regulation of OPG expression is associated not only with the activation of osteoblastogenesis, but also the inhibition of osteoclast differentiation due to the suppression of RANKL signaling [299]. The inhibition of RANKL signaling with the down-regulation of osteoclastogenesis may also be mediated by the anti-inflammatory effect of Sr [300] and Sr ranelate-induced activation of calcium-sensing receptor [301]. In addition, the up-regulation of LRP6/β-catenin/OPG signaling may also underlie the inhibitory effect of Sr on osteoclastogenesis [302].

To date, the existing epidemiological studies and clinical trials demonstrate that Sr as Sr ranelate induce significant antiosteoporotic effects characterized by increased BMD and reduction in fracture risk, also promoting bone regeneration, although excessive Sr ranelate intake may be associated with a higher incidence of CVD. Clinical effects of Sr are achieved through promotion of osteoblastogenesis and osteoblast activity through the up-regulation of BMP-2 signaling, the activation of the Wnt/β-catenin and TGF-β/Smad pathways, as well as MAPK/ERK activation, the induction of AMPK-activated autophagy, and other pathways. At the same time, the osteogenic effect appears to be dose-dependent with the inhibition of osteoblast activity at high-dose Sr exposure. In addition, Sr is also capable of reducing bone resorption by inhibiting RANKL-induced osteoclast differentiation and the up-regulation of OPG signaling.

## 10. Silicon (Si)

Congruent with the role of Si in connective tissue functioning [303], several studies demonstrated a significant positive relationship between dietary Si intake and bone regeneration [304]. Specifically, in the Framingham Offspring cohort of 1251 men and 1596 women, Si intake was shown to be positively associated with hip BMD in men and premenopausal women, but not postmenopausal ones [305]. The interaction between Si and estrogen status was shown to have a significant impact on BMD, characterized by a positive association between Si intake and BMD only in estrogen-replete women from the UK [306]. A 3-month Si supplementation was shown to reduce oxidative stress and bone resorption in Italian menopausal osteopenic women [307].

In contrast, Si overexposure may have adverse effects on bone quality markers. Specifically, occupational Si exposure in Turkish stone carvers or quartz miners is associated with reduced 25-hydroxycalciferol levels and BMD [308], being in agreement with laboratory studies in a rat model of silicosis [309]. At the same time, increased intake of Si with artesian drinking water for 12 weeks was not associated with alterations in bone resorption [310].

Laboratory in vivo studies also demonstrated the beneficial effect of nutritional Si on bone health. In Si-supplemented rats serum, Si levels were found to correlate significantly with serum osteocalcin levels and BMD, although this effect was observed only in female but not male rats [311]. Si also induced the antiosteoporotic effect in Ca-deficient ovariectomized rats through the inhibition of bone resorption [312]. Correspondingly, Si increased BMD in ovariectomy-induced osteoporosis [313], while bioactive silica nanoparticles reversed ageing-associated bone loss [314].

In vitro studies demonstrated that Si promotes osteoblast differentiation [315] via the up-regulation of BMP-2 signaling [316] and the subsequent activation of the BMP-2/Smad1/5/RUNX2 signaling pathway, resulting in increased osteocalcin [317] and type I collagen expression by osteoblasts [318]. Si-induced increase in osteoblast differentiation is also associated with the up-regulation of and stimulation of gap junction communication [319].

It has also been demonstrated that Si promotes Wnt/β-catenin signaling through the up-regulation of Lrp5 and the down-regulation of DKK1 expression, altogether resulting in increased osteoblast expression [320]. Being in agreement with the earlier observed tight interplay between PI3K-AKT-mTOR and Wnt signaling [321], Si-induced osteogenesis was shown to be dependent on the activation of the PI3K–Akt–mTOR pathway [322]. The protective effect of Si against glucocorticoid-induced osteoporosis and osteocyte apoptosis was also shown to be mediated by increased Akt phosphorylation [323].

The up-regulation of p38 MAPK [324] and ERK signaling [325] was also associated with the osteogenic effect of Sr. In addition, the modulation of ERK signaling may also contribute to the stimulation of osteoblast differentiation through the up-regulation of autophagy [326].

Si was shown to up-regulate the expression of miR-146a, which inhibits TNFα-induced activation of NF-κB, thus promoting osteoblast differentiation and exerting osteoclast-inhibiting activity [327].

Osteoclasts should also be considered as targets for biological effects of Sr in bone. Si may inhibit osteoclastogenesis through the suppression of M-CSF and RANKL expression [328], thus resulting in reduced bone resorption [329]. It has been demonstrated that the down-regulation of NF-κB activation may be responsible for both inhibitory effects of Si on osteoclast-dependent bone resorption and an increase in osteoblast activity [330]. Si-induced inhibition of RANKL signaling is associated with the down-regulation of NFATc1 and other osteoclast-specific genes, which may underlie the protective role of ortho-silicic acid in ovariectomy-induced bone loss [331]. The results of another study demonstrate that Si was also shown to increase OPG expression without any significant impact on the RANKL expression level, thus promoting a shift to osteoblastogenesis from osteoclastogenesis [332]. At the same time, certain studies demonstrate that Si may induce a stimulatory effect on osteoclast activity [333].

Taken together, the existing data demonstrate that dietary Si intake is associated with improved BMD, although at higher doses, including occupational exposure cases, Si may induce adverse effects on bone quality. Epidemiological and clinical findings generally corroborate the obtained data on the protective effect of Si in animal models of osteoporosis. The up-regulation of BMP-2 and Wnt/β-catenin signaling was shown to mediate the osteogenic effect of Si. The latter was also shown to be associated with the activation of PI3K/Akt and MAPK/ERK pathways. In addition to the promotion of osteoblastogenesis, Si was shown to increase OPG expression and inhibit M-CSF and RANKL-induced osteoclast differentiation with the down-regulation of NF-κB signaling. These findings demonstrate that Si should be considered protective against osteoporosis by promoting bone formation and reducing its resorption.

## 11. Concluding Remarks

The existing data demonstrate a significant association between essential trace element and mineral body burden and the risk of osteoporosis. At the same time, the effect on bone health appears to be dose-dependent, with low doses promoting osteogenic effects, whereas high doses exert opposite effects that may promote bone resorption and impaired bone formation. Such a U-shaped relationship between the dose and osteogenic response was especially profound in the case of Fe, Cu, F, and Sr.

Nonetheless, it is noteworthy that some studies have failed to reveal significant associations between trace element and mineral intake and osteoporosis despite their role in bone physiology and the relationship between trace element and mineral status and bone health. These findings may be indicative of the limited role of malnutrition, and the primary role of impaired metal homeostasis in osteoporosis in well-nourished populations. Specifically, the causal factors of osteoporosis may significantly modulate trace element and mineral metabolism. Ageing is associated with the significant modulation of transport and metabolism of Fe [334], Zn [335], Cu [336], Se [337], and Mg [338], resulting in its deficiency. In turn, recent findings demonstrate that menopause is associated with altered trace element and mineral metabolism [339] including Mg [338], Se [340], Fe [341], Zn [342], and Cu [343] at least due to the deficiency of the impact of estrogen on trace element and mineral transport [338,344,345,346]. Hypothetically, in view of the role of trace elements and minerals in the regulation of bone physiology, alterations in their metabolism along the ageing axis or menopause should be considered as potential additional factors contributing to the pathogenesis of senile and postmenopausal osteoporosis.

In addition, given the distinct effects of trace elements and minerals on bone physiology [347], elemental interactions may significantly modulate the relationship between particular trace elements and minerals and osteoporosis. Specifically, high-dose Zn intake was shown to promote Mg excretion in osteoporotic women [348]. An antagonistic relationship between Cu and Zn, as well as Fe and Zn, may also have a significant effect on bone tissue metabolism and modulate the risk of osteoporosis [349]. Both Se [350] and Zn [351] were shown to antagonize adverse effects of fluoride exposure in the organism. In addition, several interactions may potentiate the effects of particular trace elements and minerals in bones through the positive modulation of bioavailability as observed for Cu and Fe [352], Zn and Se [353], Fe and Co [354]. Finally, a number of elements including Fe [355], Zn [356], and Mg [357] significantly modulate Ca^2+^ metabolism, which may also be considered as a potential mechanism mediating the role of altered trace element and mineral metabolism in osteoporosis. Therefore, the role of particular elements in bone metabolism and osteoporosis pathogenesis may also be mediated by other trace elements and minerals that modify their bioavailability and handling.

The existing laboratory data demonstrate that essential trace elements and minerals exert a significant modulatory effect on bone physiology by regulating bone formation and bone resorption. Physiological and nutritional levels of trace elements and minerals promote osteogenic differentiation through a plethora of mechanisms, including the up-regulation of BMP-2 and Wnt/β-catenin signaling, as well as the stimulation of TGF1β/Smad, PI3K/Akt/GSK3β, and MAPK/ERK pathways, also protecting osteoblasts from oxidative stress, ferroptosis, endoplasmic reticulum stress, mitochondrial dysfunction, and apoptosis. Recent findings demonstrate a significant role of miRNA and epigenetic factors in regulating the osteogenic effects of micronutrients. In addition, trace elements and minerals contribute to a reduction in bone resorption through the inhibition of RANKL-induced osteoclastogenesis, stimulation of OPG signaling, as well as inhibition of the inflammatory response.

It appears that nutritional or ageing-associated deficiency of essential elements including Mg, Se, Fe, Zn, and Cu results in the alteration of the above-mentioned mechanisms, resulting in the inhibition of osteogenesis along with the promotion of osteoclastogenesis with subsequent bone resorption. In turn, the improvement of body burden of essential trace elements and minerals results in physiological bone remodeling and reduced osteoporosis risk. Although the relevance of the nutritional deficiency of F, Sr, and Si in humans is rather questionable, it is proposed that these elements may promote osteogenic response at nutritional and supranutritional doses. In addition, excessive intake of essential elements, as well as F, Sr, and Si overload, may induce adverse effects on bone health. Correspondingly, the results from recent meta-analyses demonstrate that intake/supplementation with Zn, Mg, F, and Sr improve bone quality, thus exerting antiosteoporotic effects.

Based on the recent findings, it is proposed that an improvement of essential trace element and mineral nutrition, especially Zn and Mg, may be considered as the primary approach for the improvement of bone health in malnourished populations as well as subjects with high risk of essential element deficiency. In turn, in subjects with low risk of essential trace element and mineral deficiency, supplementation with F or Sr could be considered as a potential preventive strategy to reduce the risk of osteoporosis. At the same time, in view of their narrow therapeutic window, F and Sr supplementation should be performed with caution due to the high risk of overexposure.

Taken together, the existing data demonstrate that an improvement in trace element and mineral status by alleviating its dietary insufficiency and overload, as well as an improvement of its metabolism, can contribute to the prevention of osteoporosis. However, further studies are required for the investigation of the underpinning mechanisms of micronutrients in bone physiology, and the estimation of the efficiency of micronutrient supplementation in improving bone quality in osteoporotic patients as well as for prevention.

## Data Availability

Not applicable.

## Abbreviations

Akt—Akt serine/threonine kinase; AMPK—AMP-activated protein kinase; ATF4—activating transcription factor 4; BMD—bone mineral density; BMMSCs—bone marrow mesenchymal stromal cells; BMP—bone morphogenetic protein; Bnip3—Bcl-2 interacting protein 3; CA II—carbonic anhydrase II; CALCA—calcitonin-related polypeptide alpha; cAMP—cyclic adenosine monophosphate; CaSR—calcium-sensing receptor; CHOP—CCAAT/enhancer-binding protein (C/EBP) homologous protein; COX—cyclooxygenase; CREB—cAMP response element-binding protein; CVD—cardiovascular disease; DUSP14—dual specificity phosphatase 14; eIF2α—eukaryotic initiation factor-2α; ERK extracellular signal-regulated kinase; FOXO3—Forkhead box O3; GPX—glutathione peroxidase; GSK3β—glycogen synthase kinase-3 beta; HIF-1α—hypoxia-inducible factor 1-alpha; HO-1—heme oxygenase 1; HUVECs—human umbilical vein endothelial cells; IFNγ—interferon gamma; IGF-1—insulin-like growth factor 1; IGF-1R—IGF-1 receptor; IL—interleukin; iNOS—inducible nitric oxide (NO) synthase; IRE—iron-responsive element; IκB—inhibitor of nuclear factor kappa B; LPO—lipid peroxidation; LPS—lipopolysaccharide; LRP—low-density lipoprotein receptor-related protein; MCP-1—monocyte chemoattractant protein-1; M-CSFR—macrophage-colony stimulating factor receptor; METAP2—methionyl aminopeptidase 2; miRNA—MicroRNA; MITF—microphthalmia-associated transcription factor; MLH1—MutL protein homolog 1; MMP—matrix metalloproteinase; mTOR—mammalian target of rapamycin; NFATc1—nuclear factor of activated T-cells; NF-κB—nuclear factor κB; NHANES—National Health and Nutrition Examination Survey; NPs—nanoparticles; OPG—osteoprotegerin; PCK—phosphoenolpyruvate carboxykinase; PI3K—phosphatidylinositol-3 kinase; PKA—protein kinase A; PTEN—phosphatase and tensin homolog; PTH—parathyroid hormone; PTHrP—PTH-related peptide; Rab7—Ras-related protein 7; RANK receptor activator of nuclear factor-kappa B; RANKL—receptor activator of nuclear factor-kappa B ligand; RAS—rat sarcoma viral oncogene homolog; ROS—reactive oxygen species; Runx2—runt-related transcription factor 2; SELENOP—selenoprotein P; SELENOW—selenoprotein W; SIRT1—sirtuin 1; SrRa—strontium ranelate; STAT3—signal transducer and activator of transcription 3; TGF-β1—transforming growth factor beta; TLR4—toll-like receptor 4; TNF—tumor necrosis factor; TRAF6—TNF receptor-associated factor 6; TRAP—tartrate-resistant acid phosphatase; TXNRD—thioredoxin reductase; TβR2—TGF-β receptor 2; VEGF—vascular endothelial growth factor; YAP—Yes-associated protein; Zfp—zinc finger protein.
